# Apoptosis inhibitor of macrophage (AIM/CD5L) as a potential immunomodulator in feline injection-site sarcoma: a hypothesis on tumor–macrophage interactions

**DOI:** 10.1080/01652176.2026.2706036

**Published:** 2026-08-02

**Authors:** Julia Gilbert, Gyula Balka, Míra Mándoki, Anna Szilasi

**Affiliations:** a University of Veterinary Medicine, Budapest, Department of Pathology, Budapest, Hungary

**Keywords:** Apoptosis inhibitor of macrophage (AIM/CD5L), feline injection-site sarcoma (FISS), tumor microenvironment, tumor-associated macrophages, inflammation-driven cancer, macrophage polarisation, complement activation, comparative oncology

## Abstract

AIM (apoptosis inhibitor of macrophage), also known as CD5L, is a macrophage-derived scavenger protein with broad immunomodulatory roles in mammals. Secreted by tissue macrophages, AIM circulates bound to IgM pentamers, which stabilize the protein in blood; free AIM is released during inflammation to influence immune signaling and cellular homeostasis. (Yang et al. 2023). In cats, AIM exhibits distinctive structural and biochemical properties, existing as both a three-domain (37 kDa) and a four-domain (45 kDa) variant generated through exon 3 duplication (Evangelista et al. 2025), and binding IgM approximately 1000-fold more tightly than murine AIM due to a species-specific positively charged cluster in its third SRCR domain (Miyazaki et al., 2018). These differences suggest feline AIM may function distinctly from human/mouse AIM. Feline injection-site sarcomas (FISS) arise in the context of chronic inflammation and frequently contain abundant tumor-associated macrophages (TAMs)associated with tumor aggressiveness and progression (Gomes et al., 2025). Given AIM’s macrophage origin and its established roles in inflammatory regulation, autophagy signaling, and complement-mediated tumor targeting, we propose a directional hypothesis: that the high-affinity IgM-binding of feline AIM constrains free AIM availability in vivo, shifting the functional balance away from complement-mediated tumor cytotoxicity and toward macrophage-intrinsic M2-polarizing pathways, thereby promoting an immunosuppressive tumor microenvironment that contributes to FISS progression. Exploring AIM in this context may provide new perspectives for investigation in feline tumor immunology.

## Simple summary

Feline injection-site sarcoma (FISS) is an aggressive cancer that develops in cats at sites of chronic inflammation, most commonly following injections or vaccinations. These tumors are characterized by a strong inflammatory component and the presence of large amounts of immune cells, particularly macrophages, which can influence tumor development and progression. Apoptosis inhibitor of macrophage (AIM), also known as CD5L, is a protein produced by macrophages that plays an important role in regulating immune responses. AIM participates in several biological processes, including control of inflammatory signaling, regulation of macrophage survival and activity, induction of autophagy, and activation of the complement system.

Cats possess unique features in their AIM protein compared with other species, including structural variants and an unusually strong interaction with immunoglobulin M (IgM), which results in relatively high levels of AIM circulating in feline blood. Despite its central role in immune regulation, AIM has not yet been investigated in feline cancers.

In this article, we review current knowledge about AIM structure and function and propose a hypothesis that AIM may influence the immune microenvironment of feline injection-site sarcoma. Specifically, AIM could contribute to the regulation of macrophage activity and inflammatory pathways within tumors. Exploring this possibility may improve understanding of the immune mechanisms underlying FISS and highlight new directions for research into inflammation-driven cancers in cats.

## 1. Introduction

AIM (apoptosis inhibitor of macrophage, CD5L) is a soluble protein secreted primarily by tissue-resident macrophages. It contains three scavenger receptor cysteine-rich (SRCR) domains and circulates in blood, bound to IgM pentamers. This IgM–AIM complex prevents renal clearance, maintaining high serum AIM levels (≈5 µg/mL in humans) (Yang et al. 2023). When dissociated from IgM, AIM can regulate immune responses: promotes clearance of cellular debris, modulates cytokine production, and influences macrophage survival and phenotype (Sanjurjo et al. 2015; Kim et al. 2021; Yang et al. 2023). In particular, AIM has been implicated in anti-inflammatory signalling via an IL-10–STAT3 feedback loop and in autophagy induction through CD36 (Sanjurjo et al. 2015; Kim et al. 2021). Notably, experimental studies in mice have shown that macrophage-derived AIM can deposit on hepatocellular carcinoma (HCC) cells, activate complement, and induce tumour cell destruction (Yang et al. 2023), suggesting a potential role in tumour immunity.

Feline injection-site sarcomas (FISS) are aggressive mesenchymal tumours arising at sites of chronic injection-associated inflammation, commonly following vaccination or other subcutaneous injections. Histopathologically, FISS often contains extensive infiltrates of macrophages, lymphocytes, and giant cells, with areas of necrosis (Sanjurjo et al. 2015). Recent immunohistochemical studies found that tumour-associated macrophage (TAM) density in FISS correlates with tumour aggressiveness (higher TAM infiltration was associated with increased necrosis, mitotic index, and poorer differentiation) (Gomes et al. 2025). Similar associations between TAM infiltration and tumour biology have been described in other veterinary sarcomas, including canine visceral hemangiosarcoma, further supporting a conserved role for macrophages in the progression of mesenchymal tumours (Kerboeuf et al. 2024). These observations highlight macrophages as key regulators of the inflammatory tumour microenvironment in FISS. Despite the central role of macrophages in this disease, the molecular factors that regulate macrophage behaviour in FISS remain incompletely understood. AIM biology has not yet been investigated in feline neoplasia; however, given its macrophage origin and its established roles in immune regulation, chronic inflammation, and tumour immunity in other systems, it represents a plausible candidate mediator within this context.

In this hypothesis-driven review, we synthesise current knowledge of AIM structure and biology, with particular attention to the distinctive features of feline AIM, including structural variants and unusually strong IgM binding. We then integrate these findings with existing knowledge of FISS pathogenesis to propose a conceptual framework in which AIM may influence macrophage activity and immune signalling within the FISS tumour microenvironment. By outlining this theoretical model, we aim to highlight potential avenues for future investigation into the immunological mechanisms underlying inflammation-driven sarcomas in cats.

This review was conducted as a narrative review, a format selected because the absence of prior studies directly investigating AIM in feline neoplasia precluded the systematic inclusion and quantitative synthesis of a defined study population, as would be required by scoping or systematic review design. Literature searches were conducted across two parallel streams. For AIM/CD5L biology, searches were performed in PubMed and Google Scholar using the terms “AIM”, “CD5L”, “apoptosis inhibitor of macrophage”, “SRCR domain”, “IgM-AIM complex”, and combinations thereof, with no date restriction, to capture the full available evidence base on AIM structure, function, and species-specific characteristics. For FISS pathogenesis and tumour microenvironment, searches used terms including “feline injection-site sarcoma”, “feline vaccine-associated sarcoma”, “FISS tumour microenvironment”, “tumour-associated macrophages feline”, and “feline sarcoma inflammation”. Results from these two streams were integrated manually, with mechanistic connections identified through the authors' synthesis rather than through a predefined mapping protocol.

A central interpretive challenge in this review is the asymmetry of available evidence across species. The functional pathways through which AIM influences macrophage behaviour, including IL-10/NLRP3 inflammasome regulation, CD36-mediated autophagy induction, and complement-dependent cytotoxicity, have been characterised almost exclusively in murine and, to a lesser extent, human experimental systems. Feline-specific data are currently limited to structural and biochemical characterisation of the AIM protein and its IgM interaction, and to one recent study examining AIM variants in the context of feline chronic kidney disease (Evangelista et al. 2025). In the absence of direct feline functional data, mechanistic inferences in this review are explicitly extrapolated from murine and human models and are presented as hypotheses rather than established facts. Where species-specific differences in AIM biology are known, particularly the feline exon 3 duplication and the high-affinity IgM interaction, their potential consequences for pathway function are discussed as modifying factors that may alter how murine findings translate to the feline context. Readers should interpret the proposed mechanistic model with this evidentiary asymmetry in mind.

## 2. Background and rationale

Feline injection-site sarcomas (FISS) represent a unique model of inflammation-associated tumorigenesis in which persistent inflammatory stimulation and dysregulated wound-healing responses are thought to drive neoplastic transformation. Although their exact pathogenesis remains incompletely understood, persistent inflammatory stimulation and dysregulated wound-healing responses are believed to play central roles in tumour development. Histologically, FISS lesions are characterised by proliferating fibroblast-like neoplastic cells embedded within a dense inflammatory microenvironment that frequently includes macrophages, lymphocytes, and multinucleated giant cells, often accompanied by areas of necrosis and fibrosis. These tumours exhibit locally invasive growth and high recurrence rates following surgical excision. Increasing evidence suggests that immune cells within the tumour microenvironment, particularly macrophages, contribute to disease progression by shaping inflammatory signalling and tissue remodelling processes associated with tumour development (Sanjurjo et al. 2015; Gomes et al. 2025).

Given the central role of macrophages in FISS pathology, molecules that regulate macrophage survival and immune signalling may influence disease dynamics. One such molecule is the apoptosis inhibitor of macrophage (AIM), encoded by the CD5L gene. AIM is a secreted protein of approximately 40 kDa composed of three tandem scavenger receptor cysteine-rich (SRCR) domains. In macrophages, its transcription is regulated by nuclear receptors such as liver X receptor (LXR) and retinoid X receptor (RXR), as well as transcription factors including MafB. Once secreted, AIM circulates in the bloodstream tightly bound to the Fc portion of IgM pentamers, which protects the protein from renal clearance and maintains relatively high systemic concentrations under physiological conditions. In this IgM-bound state, AIM is largely inactive; however, during inflammation or tissue injury, AIM can dissociate from IgM, potentially through proteolytic mechanisms, allowing it to interact with target cells and exert immunomodulatory effects (Yang et al. 2023). Structural studies have shown that the AIM–IgM interaction involves multiple SRCR domains. Mapping analyses suggest that AIM forms a disulphide linkage with a cysteine residue within the IgM Fc region, while electrostatic interactions between the positively charged surface of AIM’s third SRCR domain and the Cμ4 domain of IgM further stabilise the complex (Miyazaki et al. 2018; Yang et al. 2023).

Through this binding mechanism, circulating AIM can remain in a “stand-by” state and become functionally available when inflammatory signals disrupt the complex.

### 2.1. Species differences—Feline AIM variants and IgM binding

Although the basic architecture of AIM is conserved across mammals, important species-specific differences exist. In humans and mice, AIM is a three-domain protein of approximately 37–42 kDa encoded by six exons. Domestic cats, however, express two distinct AIM variants: the canonical three-domain form and a larger four-domain protein generated through duplication of exon 3 within the CD5L gene. This duplication results in an additional SRCR1 domain, producing a 45 kDa protein variant. Genomic analyses indicate that this allele is common in cats, with approximately 60% of individuals carrying the duplication. While the functional consequences of this additional domain remain unclear, structural alterations of scavenger receptor proteins often influence ligand binding and immune signalling, suggesting that feline AIM variants may possess distinct biological properties (Evangelista et al. 2025).

Another notable feature of feline AIM is its exceptionally strong affinity for IgM. Biochemical studies have demonstrated that the dissociation constant (K_D) of feline AIM for IgM-Fc is approximately 6 × 10^−9^ M, compared with roughly 6 × 10^−6^ M for mouse AIM, a nearly 1000-fold difference in binding strength. This high affinity is attributed to a cluster of positively charged amino acid residues located within the SRCR3 domain of feline AIM that is absent in the corresponding regions of human and murine proteins. Experimental mutation of these residues reduces IgM binding affinity, confirming their importance in stabilising the complex. The unusually tight association between feline AIM and IgM has functional implications for inflammatory responses. Because feline AIM remains strongly bound to IgM even during tissue injury, the protein may be less readily mobilised than in other species.

Experimental observations in models of feline kidney injury suggest that this limited dissociation can impair the availability of free AIM required for efficient clearance of cellular debris, potentially contributing to species-specific susceptibility to certain inflammatory conditions. Consistent with this binding behaviour, circulating AIM concentrations in cats are relatively high, averaging approximately 21 µg/mL in serum, substantially higher than levels reported in humans or mice, and closely correlating with IgM abundance in the bloodstream (Miyazaki et al. 2018; Chen et al. 2024).

Taken together, these structural and biochemical differences suggest that feline AIM may behave uniquely during inflammatory responses, and their functional consequences for each of the major AIM-associated pathways merit explicit consideration. With respect to complement activation, the high-affinity IgM sequestration of feline AIM has a direct and quantitatively important implication: the concentration of free, dissociated AIM available to deposit on cell surfaces is likely substantially lower in cats than in mice or humans under equivalent inflammatory conditions. In murine models, complement-mediated tumour cell lysis by AIM depends on sufficient free AIM accumulating on tumour cell surfaces to inactivate complement regulatory proteins (Yang et al. 2023). If feline AIM dissociates from IgM far less readily, as its binding kinetics strongly suggest, then the threshold concentration of free AIM required for effective complement deposition may rarely be reached in feline tissues, even during active inflammation. This represents a fundamental species-specific constraint on AIM's tumoricidal function that does not apply in the murine systems from which most complement-related AIM data are derived.

The implications for macrophage regulation are more nuanced. The IL-10/NLRP3 inflammasome suppression pathway and the CD36-mediated autophagy pathway through which AIM modulates macrophage behaviour are both dependent on AIM interacting with macrophage surface receptors, functions that require free rather than IgM-bound AIM. However, because tumour-associated macrophages are themselves a local source of AIM secretion, the relevant pool of free AIM for macrophage-intrinsic signalling may be the locally secreted fraction rather than systemically circulating AIM. TAMs within the FISS microenvironment could therefore generate sufficient paracrine free AIM to engage these pathways regardless of the systemic IgM sequestration constraint (Maehara et al. 2014). This distinction between systemic circulating AIM and locally secreted AIM is mechanistically important: it suggests that macrophage-intrinsic IL-10 and autophagy signalling may be relatively preserved in the FISS microenvironment, even as complement-activating free AIM remains constrained by the high systemic IgM affinity (Miyazaki et al. 2018; Yang et al. 2023).

For tumour immunity overall, the net consequence of these two considerations is a predicted functional asymmetry: the macrophage-polarising pathways of AIM are more likely to be operative in the FISS microenvironment than its complement-dependent cytotoxic pathway. Understanding this asymmetry, and its derivation from feline-specific IgM-binding biochemistry, is central to the hypothesis developed in this review.

Because FISS arises in the context of chronic inflammation characterised by substantial macrophage infiltration, these distinctive features of feline AIM raise the possibility that the protein could influence immune signalling within the tumour microenvironment. Understanding how AIM functions in the feline immune system, therefore, represents an important step toward evaluating its potential relevance to inflammation-associated tumours such as FISS.

We hypothesise that the exceptionally high affinity of feline AIM for IgM, approximately 1000-fold greater than in mice, constrains the systemic release of free, bioactive AIM, thereby limiting its complement-activating potential on tumour cell surfaces. As a consequence, we propose that within the FISS tumour microenvironment, AIM/CD5L functions predominantly through its macrophage-intrinsic signalling pathways, specifically IL-10/NLRP3 inflammasome suppression and CD36-mediated autophagy induction, to promote M2-like polarisation of tumour-associated macrophages and sustain an immunosuppressive microenvironment that supports tumour progression (Fujiwara et al. 2021). This directional hypothesis is grounded in the species-specific biochemistry of feline AIM and predicts that AIM acts as a pro-tumorigenic, rather than tumoricidal, mediator in the context of FISS.

## 3. Functional pathways of AIM relevant to tumour immunity

AIM participates in several immunological pathways that have the potential to influence tumour biology. These mechanisms include regulation of inflammatory cytokine signalling, modulation of macrophage autophagy, and activation of complement-mediated cytotoxicity against tumour cells. Although these pathways have primarily been described in non-feline systems, their mechanistic implications provide a conceptual framework for considering how AIM could affect inflammation-associated tumours. Importantly, as discussed in Section 2.1, the high-affinity IgM sequestration characteristic of feline AIM is expected to differentially constrain these pathways: complement activation, which requires free AIM accumulation on cell surfaces, is likely more limited in cats than in murine models, whereas macrophage-intrinsic IL-10 and autophagy signalling, potentially sustained by locally secreted TAM-derived AIM, may remain functionally relevant within the FISS tumour microenvironment. This species-specific modification of pathway activity is incorporated into the mechanistic discussion that follows.

### 3.1. IL-10/inflammasome pathway

AIM is known to be involved in the IL-10 anti-inflammatory circuit. In murine macrophages, IL-10 stimulation upregulates AIM expression via STAT3 binding to the AIM promoter. AIM in turn is required for IL-10’s ability to suppress the NLRP3 inflammasome. AIM-knockout macrophages do not show the normal IL-10–induced reduction in caspase-1 activation and IL-1β/IL-18 secretion. Conversely, adding recombinant AIM to macrophages mimics IL-10’s effect by inhibiting ASC speck formation and cytokine release (Kim et al. 2021). Thus, AIM acts downstream of IL-10 to dampen pro-inflammatory inflammasome signals. In a tumour setting, such an IL-10–AIM loop would favour an anti-inflammatory (M2-like) macrophage phenotype and limit TLR/NLR-mediated inflammation.

Through this mechanism, AIM functions as a downstream effector of IL-10 signalling and contributes to the regulation of inflammatory responses. Within a tumour microenvironment, such an IL-10–AIM axis could favour the development of an anti-inflammatory macrophage phenotype commonly associated with tumour-associated macrophages (TAMs). This shift toward an IL-10-dominant signalling environment may limit excessive inflammasome activation while promoting immune regulatory pathways that influence tumour–immune interactions.

In the specific context of FISS, this pathway may be relevant to two prominent histological features: the formation of multinucleated giant cells and the development of a fibrotic stromal reaction. Giant cell formation is driven in part by macrophage fusion, a process that is promoted by IL-10 and suppressed by sustained NLRP3 inflammasome activity (Gao et al. 2025). If AIM dampens inflammasome-driven pro-inflammatory signalling in FISS-associated macrophages, this could create a permissive environment for the macrophage fusion events that give rise to the giant cells frequently observed in these tumours (Ahmadzadeh et al. 2022). Similarly, the fibrotic remodelling that characterises FISS stroma may be partly downstream of IL-10-polarised TAMs, which are known to promote collagen deposition and tissue remodelling (Steen et al. 2020). We therefore hypothesise that the IL-10–AIM axis may contribute to both giant cell accumulation and stromal fibrosis in FISS, two pathological features that are currently unexplained at the molecular level.

### 3.2. Autophagy and cytokine modulation via CD36

In addition to cytokine signalling, AIM has been shown to regulate macrophage autophagy through interaction with the scavenger receptor CD36. Human AIM/CD5L binds the scavenger receptor CD36 on macrophages, activating class III phosphatidylinositol 3-kinase (PIK3C3/VPS34) and inducing autophagy. Macrophages exposed to AIM exhibit increased levels of the autophagy marker LC3-II and enhanced autophagic activity even in the absence of additional inflammatory stimuli. This AIM-induced autophagy is accompanied by changes in cytokine production. Macrophages pretreated with AIM and subsequently stimulated with microbial ligands such as lipopolysaccharide (LPS) or Pam3CSK4 produce reduced amounts of the pro-inflammatory cytokines TNF and IL-1β while increasing secretion of IL-10. Importantly, these effects are abolished when CD36 expression is suppressed, indicating that the AIM–CD36 interaction is essential for this regulatory pathway (Sanjurjo et al. 2015).

Beyond its effects on cytokine production, AIM-induced autophagy has also been linked to macrophage polarisation. AIM/CD5L can promote differentiation toward an M2-like phenotype through autophagy-dependent upregulation of transcriptional regulators such as inhibitor of DNA binding 3 (ID3), further supporting its role in shaping macrophage functional states. This suggests that AIM-mediated autophagy is not only a degradative process but also a mechanism of immune reprogramming (Maehara et al. 2014; Sanjurjo et al., 2018).

Within tumour environments, macrophage autophagy has been implicated in the functional reprogramming of TAMs. AIM-mediated activation of this pathway could therefore influence macrophage polarisation, promoting a regulatory or tissue-remodelling phenotype characterised by increased IL-10 production and diminished pro-inflammatory signalling.

A particularly relevant consideration for FISS is the extensive necrosis that characterises these tumours histologically. Necrotic tumour microenvironments impose significant metabolic stress on infiltrating immune cells, including nutrient deprivation, hypoxia, and exposure to oxidative damage (Yee and Li 2021). Autophagy is an established cytoprotective mechanism under such conditions, enabling cells to recycle intracellular components and maintain viability in resource-limited environments (Dikic and Elazar 2018). We hypothesise that AIM-induced autophagy via CD36 could enhance the survival of TAMs within the necrotic zones of FISS, thereby sustaining macrophage infiltration even in areas of advanced tumour necrosis (Sanjurjo et al. 2015). Given that higher TAM density in FISS has been associated with increased necrosis, mitotic index, and poorer differentiation (Kliczkowska et al. 2015; Gomes et al. 2025), the ability of AIM to support macrophage persistence through autophagy induction could represent a mechanistic link between AIM biology and the aggressive phenotype of these tumours.

### 3.3. Complement-dependent cytotoxicity on tumour cells

Beyond its effects on macrophage biology, AIM has also been shown to directly target tumour cells through complement activation. Studies in liver cancer models show that when free AIM accumulates on hepatocellular carcinoma (HCC) cell surfaces, it inactivates cell-surface complement inhibitors (like CD46/CD55) and triggers the complement cascade. This results in complement-mediated lysis of the tumour cells. The net effect is tumour prevention. In mouse models with high circulating AIM, HCC development is reduced via this mechanism. However, in normal (non-cancerous) cells, AIM deposition can be harmful (e.g. contributing to glomerular injury in IgA nephropathy) (Yang et al. 2023), but on cancer cells, it appears beneficial by promoting their clearance. Although this complement-activating function has primarily been described in hepatic tumours, the mechanism raises the possibility that AIM could similarly target other cancer cell types if present within the tumour microenvironment.

In FISS, the presence of a prominent lymphoplasmacytic infiltrate, including IgM-secreting plasma cells, is a well-documented histological feature (Sanjurjo et al. 2015; Kliczkowska et al. 2015). This is of particular relevance to complement biology, because complement activation via the classical pathway is initiated by IgM binding to antigen. If free AIM accumulates on FISS tumour cell surfaces and disrupts complement regulatory proteins, the local abundance of IgM produced by infiltrating plasma cells could amplify complement cascade activation. We therefore hypothesise that the lymphoplasmacytic infiltrate characteristic of FISS may represent not merely a bystander immune response, but a potentially functionally significant source of IgM that, in conjunction with free AIM, could contribute to complement-mediated targeting of tumour cells. Conversely, because IgM also serves as the primary carrier of circulating AIM, the local IgM pool in FISS may simultaneously act to sequester AIM in its inactive, IgM-bound state, creating a dynamic equilibrium between complement activation and AIM immobilisation (Miyazaki et al. 2018). This paradox warrants specific investigation.

Several lines of reasoning suggest that the M2-polarising arm of AIM activity is likely to predominate in FISS. First, the unusually tight binding of feline AIM to IgM means that free, bioactive AIM, the form required for complement activation on tumour cell surfaces, may be less readily available in feline tissues than in other species. The same high-affinity IgM interaction that keeps circulating AIM abundant in cats may simultaneously limit its local release in the quantities required for effective complement deposition. Second, the macrophage-rich, IL-10-conducive microenvironment of FISS, where TAM density already correlates with tumour aggressiveness and poor differentiation (Gomes et al. 2025), is consistent with a tissue environment in which immunosuppressive signalling dominates over cytotoxic immune responses. Third, AIM-induced autophagy and IL-10 production are constitutive properties of macrophage–AIM interaction, whereas complement-mediated tumour killing appears to require the accumulation of sufficient free AIM on tumour cell surfaces, a threshold that may not be met given feline AIM's sequestration by IgM.

### 3.4. Integrated mechanistic model: AIM in the FISS tumour microenvironment

The pathways described in Sections 3.1–3.3 can be integrated into a coherent, stepwise mechanistic model of how AIM may function within the FISS tumour microenvironment. This model is built on the feline-specific biochemical properties of AIM described in Section 2.1 and is intended to generate testable predictions rather than to represent established facts.

Step 1—Establishment of the inflammatory microenvironment and macrophage recruitment. Chronic injection-site inflammation in cats generates a sustained innate immune response characterised by macrophage infiltration, immunoglobulin deposition, and cytokine-driven tissue remodelling (Sanjurjo et al. 2015; Gomes et al. 2025). As the inflammatory process becomes dysregulated and neoplastic transformation occurs, tumour-associated macrophages (TAMs) accumulate within the developing FISS lesion. These TAMs, recruited from circulating monocytes and resident tissue macrophages, bring with them the capacity to both secrete and respond to AIM (Kliczkowska et al. 2015; Kim et al. 2021; Yang et al. 2023; Gomes et al. 2025).

Step 2—Local AIM secretion and IgM-binding dynamics. Within the FISS tumour microenvironment, AIM is available from two potential sources: systemically circulating AIM transported to the tumour via the vasculature, and locally secreted AIM produced by infiltrating TAMs. As discussed in Section 2.1, systemically circulating feline AIM is predominantly IgM-bound due to the exceptionally high binding affinity of the feline protein, limiting the availability of free, bioactive AIM from this source. By contrast, AIM freshly secreted by TAMs within the tumour represents a locally available paracrine pool that may engage macrophage surface receptors before systemic IgM sequestration occurs. The local IgM concentration, contributed in part by the lymphoplasmacytic infiltrate characteristic of FISS, may partially modulate this paracrine pool, but is unlikely to eliminate it entirely given the proximity of secretion to target cells (Yang et al. 2023).

Step 3—Macrophage-intrinsic pathway activation. Locally available free AIM engages two macrophage surface pathways with convergent anti-inflammatory outcomes. Through its interaction with IL-10/STAT3 signalling, AIM suppresses NLRP3 inflammasome activation, reducing caspase-1-dependent processing and secretion of IL-1β and IL-18 (Kim et al. 2021; Yang et al. 2023). Concurrently, AIM binding to CD36 activates PIK3C3/VPS34-dependent autophagy, reducing TNF and IL-1β production while increasing IL-10 secretion (Sanjurjo et al. 2015; Sanjurjo et al. 2015; Sanchez-Moral et al. 2021). Both pathways independently promote an anti-inflammatory macrophage phenotype. The convergence of inflammasome suppression and autophagy induction, both driven by locally secreted AIM, creates a reinforcing feedback loop that progressively shifts TAM polarisation toward an M2-like, immunosuppressive state. AIM-mediated upregulation of ID3 through autophagy-dependent mechanisms further consolidates this M2 polarisation at the transcriptional level (Maehara et al. 2014).

Step 4—Microenvironmental consequences of M2-polarised TAMs. M2-polarised TAMs within the FISS microenvironment exert several tumour-promoting effects. Suppressed inflammasome activity reduces the pro-inflammatory cytokine milieu that would otherwise recruit and activate cytotoxic immune cells. Elevated IL-10 production by AIM-conditioned TAMs creates a broadly immunosuppressive environment that impairs effective anti-tumour immunity (Dikic and Elazar 2018; Yee and Li 2021; Gomes et al. 2025; Kajiwara et al. 2025). Autophagy induction enhances TAM survival, particularly within the necrotic zones of FISS where metabolic stress would otherwise limit macrophage persistence. The resulting accumulation of viable, M2-polarised TAMs within the tumour is consistent with the observed association between high TAM density and poor prognosis in FISS (Gomes et al. 2025), and with the histological features of giant cell formation and stromal fibrosis that characterise these tumours.

Step 5—Constrained complement activation. In parallel, the complement-activating potential of AIM is predicted to be functionally limited in this context. Effective complement deposition on FISS tumour cell surfaces would require sufficient free AIM to accumulate locally, a condition constrained by the high-affinity sequestration of systemic AIM by IgM and potentially further limited by competition from the abundant local IgM pool contributed by infiltrating plasma cells. While complement activation cannot be excluded entirely, particularly if local inflammatory signals transiently promote AIM dissociation, it is proposed to represent a secondary and intermittent mechanism rather than a dominant anti-tumour response in this setting (Yang et al. 2023).

Predicted net outcome—The integrated model predicts that AIM functions as a net pro-tumorigenic mediator in FISS, primarily through its sustained promotion of M2-like TAM polarisation and the consequent immunosuppressive remodelling of the tumour microenvironment. This prediction is directly falsifiable: confirmation would require demonstration of AIM expression by FISS-associated TAMs, co-localisation of AIM with M2 polarisation markers in tumour tissue, and evidence of suppressed inflammasome or enhanced autophagy activity in AIM-positive macrophage populations within FISS lesions (Sanchez-Moral et al. 2021; Kajiwara et al. 2025).

## 4. Proposed hypothesis: AIM as a contributor to the FISS tumour microenvironment

Based on the biological characteristics of apoptosis inhibitor of macrophage and the inflammatory context of feline injection-site sarcomas, we propose that AIM may contribute to immune regulation within the FISS tumour microenvironment, and specifically that its dominant functional role in this setting is to promote an immunosuppressive, M2-polarised macrophage phenotype rather than to drive complement-mediated anti-tumour cytotoxicity. This directional hypothesis is grounded in three converging considerations: the species-specific sequestration of feline AIM by IgM, which limits free AIM availability (Miyazaki et al. 2018; Chen et al. 2024); the IL-10- and autophagy-mediated pathways through which AIM promotes M2 polarisation as a constitutive macrophage function; and the established association between high TAM density and poor prognosis in FISS, which is more consistent with a pro-tumorigenic immunosuppressive macrophage state than with effective anti-tumour immunity.

Chronic inflammation at injection sites in cats generates an immune milieu enriched in immunoglobulins and infiltrating macrophages, both of which represent key components of AIM biology (Sanjurjo et al. 2015; Gomes et al. 2025). Because circulating AIM is abundant in cats and tightly associated with IgM, inflammatory lesions containing macrophages and immunoglobulins could theoretically create conditions in which AIM becomes functionally engaged.

Within such an environment, AIM may originate from multiple potential sources, including systemic circulation or local macrophage secretion. Tumour-associated macrophages (TAMs), which are frequently observed in FISS, are capable of producing a wide range of immunomodulatory factors that influence tumour progression and tissue remodelling (Yang et al. 2023). Similar roles for TAMs have been described in other veterinary sarcomas, including canine visceral hemangiosarcoma, where macrophage infiltration is associated with tumour behaviour and microenvironmental regulation, supporting a broader role for macrophages in mesenchymal tumour biology (Kerboeuf et al. 2024).

**Figure 1.**
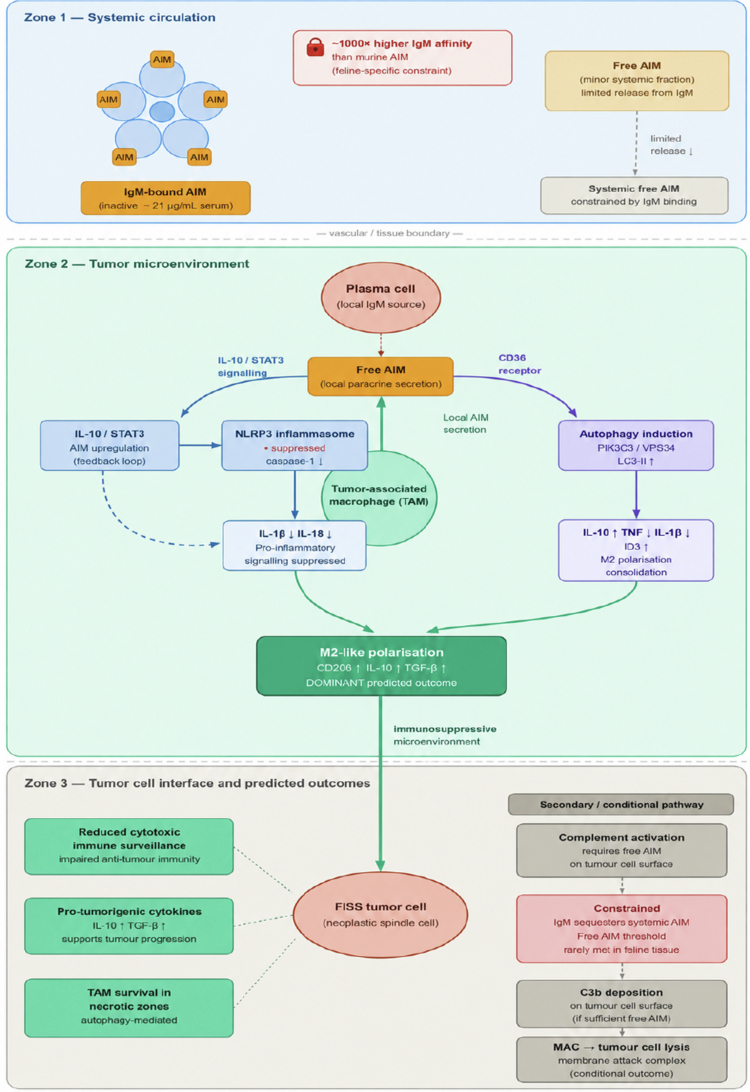
Proposed mechanistic model of AIM/CD5L function in the feline injection-site sarcoma (FISS) tumour microenvironment. Zone 1 (top) illustrates the systemic pool of AIM, predominantly sequestered in the IgM-bound inactive form due to the ~1000-fold higher IgM-binding affinity of feline AIM compared with murine AIM, limiting free AIM availability in tissue. Zone 2 (middle) depicts the tumour microenvironment, where locally secreted TAM-derived free AIM engages two convergent macrophage-intrinsic pathways: IL-10/STAT3-mediated NLRP3 inflammasome suppression and CD36-mediated autophagy induction with downstream ID3 upregulation, both promoting M2-like TAM polarisation. Local IgM contributed by infiltrating plasma cells may partially modulate the paracrine AIM pool. Zone 3 (bottom) shows the predicted tumour-promoting consequences of M2-polarised TAMs on FISS tumour cells, including suppressed cytotoxic immune surveillance and a pro-tumorigenic cytokine environment. The complement activation pathway (right) is shown as a secondary, constrained branch reflecting the limited availability of free AIM for tumour cell surface deposition under conditions of high systemic IgM sequestration. Bold arrows indicate dominant predicted pathways; dashed arrows indicate constrained or secondary pathways. (Created using BioRender software, modified by ChatGPT, OpenAI, 2026).

Given AIM’s macrophage origin and its involvement in inflammatory regulation, it is plausible that this molecule could participate in shaping immune signalling within the FISS microenvironment (Figure 1).

## 5. Conclusions, future directions, technical considerations and clinical implications for veterinary oncology

Within the context of feline injection-site sarcoma, a disease strongly associated with chronic inflammation and macrophage infiltration, AIM could plausibly influence tumour development through its effects on macrophage signalling pathways and innate immune responses. The interplay between IgM-bound and bioactive forms of AIM, together with the distinctive structural properties of the feline protein, may shape how this molecule participates in inflammatory processes occurring within tumour tissues. Although AIM has not yet been investigated in feline sarcomas, its biological functions and its prominent role in macrophage physiology make it a compelling candidate factor in the immune landscape of these tumours.

### 5.1. Future directions

Further research will be necessary to determine whether, and in what capacity, AIM participates in the pathogenesis and immune regulation of feline injection-site sarcoma. Based on the mechanistic model proposed in Section 3.4, we outline a three-tier experimental framework organised from observational to mechanistic investigation.

Tier 1—Tissue-based expression studies. The most immediately feasible first step is immunohistochemical evaluation of AIM protein expression in archived FISS tissue sections. Such studies should include antibody co-localisation to establish whether AIM-positive cells correspond to TAM populations, ideally using established macrophage markers such as Iba-1 or CD68 (Gomes et al. 2025) alongside AIM staining, and should compare AIM distribution across the tumour core, invasive margin, and peritumoral inflammatory zones. Given the mechanistic model proposed here, particular attention should be paid to AIM expression within necrotic zones and in regions of high TAM density. Parallel IgM staining would allow preliminary assessment of whether AIM co-localises with IgM deposits, providing an indirect tissue-level surrogate for distinguishing IgM-bound from free AIM. Control tissues should include non-neoplastic feline injection-site inflammatory lesions and histologically normal subcutaneous tissue to establish baseline AIM expression in the absence of neoplastic transformation. CD5L gene variant analysis in matched tumour and germline DNA from the same individuals could additionally explore whether the exon 3 duplication allele is associated with differences in AIM expression level or distribution within tumours.

Tier 2—Circulating and tissue AIM quantification. To evaluate the systemic AIM pool and its relationship to FISS, quantification of total serum AIM and, critically, the fraction of free versus IgM-bound AIM in cats with FISS compared with healthy controls and cats with non-neoplastic inflammatory conditions would provide important contextual data. Sandwich ELISA using antibodies targeting epitopes accessible only in the free or IgM-bound conformations, or size-exclusion chromatography (Miyazaki et al. 2018; Chen et al. 2024) to separate AIM–IgM complexes from free AIM prior to quantification, represent methodologically tractable approaches to this question, acknowledging the technical challenges of discriminating these forms discussed below. Longitudinal sampling across treatment modalities (surgery, radiation, chemotherapy) could additionally assess whether AIM levels change in response to tumour burden reduction, providing indirect evidence for tumour-driven AIM dysregulation.

Tier 3—In vitro macrophage functional assays. Mechanistic confirmation of the proposed IL-10/inflammasome and CD36/autophagy pathways requires cell-based experimentation. Primary feline monocyte-derived macrophages, isolated from peripheral blood of healthy donor cats and differentiated under standard conditions, represent the most appropriate cellular model in the absence of validated feline macrophage cell lines. Functional assays should include: stimulation with recombinant feline AIM protein to assess effects on macrophage polarisation state using established M1/M2 marker panels (e.g. CD206, MHC II, IL-10, TNF); measurement of autophagy induction via LC3-II western blotting (Sanjurjo et al. 2015) or autophagy flux assays following AIM treatment; and NLRP3 inflammasome activity assessment via caspase-1 activation and IL-1β secretion in AIM-pretreated macrophages stimulated with canonical inflammasome activators (Kim et al. 2021). Where feasible, co-culture models incorporating feline FISS-derived tumour cells and AIM-conditioned macrophages would allow assessment of whether M2-polarised, AIM-conditioned macrophages alter tumour cell proliferation, survival, or invasive behaviour, providing a direct test of the pro-tumorigenic hypothesis. CD36 blocking experiments in AIM-stimulated macrophages would specifically assess the contribution of the autophagy pathway to observed polarisation effects.

Realising these research directions will require careful attention to several feline-specific technical challenges that remain unresolved in the field, as outlined in the following paragraph.

### 5.2. Technical considerations

Several practical obstacles must be acknowledged when designing studies to investigate AIM in feline sarcoma. First, the availability of validated, feline-reactive AIM antibodies represents a significant limiting factor for both immunohistochemical and flow cytometric approaches. To date, most anti-AIM antibodies have been developed and validated against human or murine protein, and cross-reactivity with feline AIM, particularly with the four-domain variant arising from exon 3 duplication, cannot be assumed. Researchers will likely need to validate existing reagents against recombinant feline AIM or pursue development of feline-specific antibodies, potentially using epitopes within conserved SRCR domains (Sarrias et al. 2004). Second, the lack of well-characterised feline macrophage cell lines presents a challenge for in vitro functional studies. Primary feline monocyte-derived macrophages can be obtained from peripheral blood, but their isolation, differentiation, and polarisation protocols are less standardised than those for human or murine systems, and inter-individual variability may complicate reproducibility. Immortalised feline macrophage-like cell lines, where they exist, have not been systematically validated for AIM-related signalling studies. Third, distinguishing free AIM from IgM-bound AIM in formalin-fixed, paraffin-embedded tissue sections presents a specific methodological challenge. Because feline AIM binds IgM with exceptionally high affinity, standard immunohistochemical protocols may not reliably discriminate between the two forms, and co-localisation with IgM staining alone is insufficient to determine functional state (Miyazaki et al. 2018; Evangelista et al. 2025). Approaches such as proximity ligation assays, differential extraction protocols, or mass spectrometry-based tissue proteomics may be required to address this question with adequate specificity. Recognising these constraints will be important for designing studies that generate interpretable, reproducible results and for prioritising which research questions are tractable in the near term.

### 5.3. Clinical implications for veterinary oncology

While the hypothesis presented here requires experimental validation before any clinical application can be considered, it is worth outlining the potential translational relevance of this research for practicing veterinary oncologists, particularly given the current limitations of FISS management. FISS carries a poor prognosis despite aggressive multimodal treatment combining wide surgical excision, radiation therapy, and doxorubicin-based chemotherapy, with high local recurrence rates and frequent metastatic progression reflecting the inadequacy of current treatment protocols for many patients (Woodward 2011; Martano et al. 2011; Sanjurjo et al. 2015). Novel therapeutic targets, particularly those within the tumour immune microenvironment, represent an important area of unmet clinical need.

If future studies confirm that AIM promotes M2-like macrophage polarisation and immunosuppressive signalling within the FISS tumour microenvironment, several clinically relevant implications follow. First, AIM or its downstream effectors, including IL-10, CD36-mediated autophagy signalling, or markers of M2 macrophage polarisation, could potentially serve as tissue-based biomarkers of immune microenvironment status in FISS. Immunohistochemical quantification of such markers in diagnostic biopsy specimens might contribute to prognostic stratification, complementing existing histopathological parameters such as TAM density, mitotic index, and necrosis score (Sanjurjo et al. 2015; Gomes et al. 2025). Second, if AIM-mediated pathways are found to sustain an immunosuppressive microenvironment, they could represent targets for pharmacological disruption. Strategies aimed at shifting TAM polarisation from M2 toward M1 phenotypes are under active investigation in human oncology, and insights from AIM biology could inform analogous approaches in feline patients (Martano et al. 2011). Third, the complement-activating potential of AIM, while proposed here as a secondary function in the feline context, raises the longer-term possibility that therapeutic strategies designed to promote free AIM availability or to enhance complement deposition on FISS tumour cells could augment anti-tumour immune responses. This would represent a novel immunomodulatory approach distinct from current cytotoxic protocols (Hartmann et al. 2015; Gomes et al. 2025).

It should be emphasised that these clinical implications are contingent on experimental confirmation of the proposed hypothesis and remain speculative at this stage. Nevertheless, the poor outcomes associated with FISS and the emerging interest in veterinary immuno-oncology make this a clinically meaningful research direction, and early consideration of translational endpoints in the design of future laboratory studies would maximise the relevance of findings for veterinary clinical practice.

Advancing knowledge in these areas could help determine whether AIM contributes to tumour–immune interactions in feline sarcomas, and whether its signalling pathways may represent targets for future immunomodulatory strategies. More broadly, investigating AIM biology in cats may expand current understanding of macrophage regulation in companion animal diseases and contribute to the growing field of comparative tumour immunology. By highlighting the potential intersection between AIM biology, macrophage immunology, and inflammation-driven oncogenesis, this hypothesis aims to stimulate research that bridges basic immunological science and veterinary clinical practice, ultimately contributing to improved understanding and management of one of the most challenging tumours encountered in feline oncology.
